# Dietary Intakes, Knowledge, and Perceptions of Semi-professional Rugby Athletes in Scotland

**DOI:** 10.1080/15502783.2022.2036436

**Published:** 2022-03-26

**Authors:** Sonam Hitendre, Rebecca Jordan, Christos Theodorakopoulos, Lois White

**Affiliations:** Dietetics, Nutrition & Biological Sciences, School of Health Sciences, Queen Margaret University, Edinburgh, UK

**Keywords:** Nutritional knowledge, athletes, rugby, dietary intake, performance enhancing supplements, dietary goals, nutritional strategies, perceptions, challenges

## Abstract

**Background:**

Adequate nutritional intake plays a pivotal role in optimizing performance, recovery, and body composition goals. This study aimed to investigate the dietary intakes (DIs); nutritional knowledge (NK); and attitudes, perceptions, and challenges (APC) of semiprofessional rugby players in Scotland.

**Methods:**

Dietary intakes and NK of 24 male semiprofessional rugby players of a Super6 club were evaluated using validated questionnaires. Players were categorized as having good or poor NK according to NK scores. Diet-related APCs were assessed using researcher-developed questionnaires and 1-1 semi-structured interviews.

**Results:**

Mean ± SD total NK% was poor, 53.7 ± 11.9%. The ‘Good’ NK group scored significantly higher in the Weight Management (*p* = 0.014), Macronutrients (*p* < 0.001), Micronutrients (*p* = 0.001), and Sports Nutrition (*p* < 0.001) sections. Mean DIs from food sources were 26.3 ± 9.2 kcal/kg/day energy, 1.4 ± 0.4 g/kg/day protein, and 21.7 ± 10.1 g/day fibre. Median (25th,75th) carbohydrate intake was 3.0 (2.0, 3.0) g/kg/day, and 6.3 (2.3, 10.6) units/week alcohol. Mean ± SD fat and saturated fat (SFA) % total energy intake (EI) were 36.2 ± 3.7% and 12.8 ± 1.9%, respectively, and SFA %EI exceeded recommendations (*p* < 0.001). The ‘Good’ NK group had significantly higher intakes of all macronutrients (*p* < 0.05). Total NK% positively correlated with intakes of meat (*r* = 0.556, *p* = 0.011), cereals (*r* = 0.458, *p* = 0.042), dietary fat (*r* = 0.477, *p* = 0.034), vegetables (*r* = 0.487, p = 0.030), and alcoholic beverages (*r* = 0.541, *p* = 0.014). Supplement use was 68%. Players felt diet affected performance (94%) but 31% of them were unaware of any specific nutritional strategies. A healthy diet was perceived to be ‘balanced’ with ‘variety from all food groups. Lack of time for preparation was described as the main barrier to healthy eating.

**Conclusions:**

Overall, players had poor NK, their fibre and carbohydrate intake was suboptimal, whereas saturated fat intake exceeded recommendations. Many lacked awareness of current sports nutrition guidelines. Further nutrition education may be needed to improve diet quality and aid performance goals.

## 1. Introduction

Nutrition has become increasingly recognised as a key component for optimizing sports performance and adaptations to exercise [1-3]. Despite an increasing number of athletes aiming to fuel performance through optimal nutrition, research shows that many athletes have suboptimal dietary intakes (DIs) and inadequate dietary knowledge, which may translate into poorer food choices [4,5].

Team sports have unique physiological demands [6], and despite the advances in sports nutrition, there is not an abundance of studies on the determinants of dietary practices in athletes of amateur or semi-professional leagues. Rugby is characterized by repeated alterations of high-intensity exercise placing a heavy demand on the aerobic and anaerobic energy systems [4, 7, 23]. Furthermore, dietary goals may further differ according to the position played [4,6].

Nutritional knowledge (NK) is a significant determinant of food choice [8]. Evidence shows that rugby players may place lower importance on diet compared to endurance athletes, which may lead to poorer food choices when combined with inadequate NK [4]. However, NK may not necessarily translate into better practice, and rugby athletes can have suboptimal dietary intakes, particularly of carbohydrates, regardless of NK [4]. Moreover, men’s magazines often cite ‘protein needs’ and lack messages which highlight the importance of a healthy balanced diet [5,9].

There are multiple factors that may influence dietary intakes such as cost, food preferences, convenience, availability, skills, media, education, social, cultural, and religious beliefs, among others [5,6,10,11]. Semiprofessional athletes may be presented with additional factors that may influence dietary choices such as time constraints while managing work and family commitments with busy training schedules. Furthermore, it is possible that the COVID-19 pandemic has introduced further challenges that may impact dietary intakes.

There is currently a limited number of studies on semi-professional rugby athletes. Therefore, it has not been corroborated what the actual level of knowledge and understanding of nutritional requirements is among this cohort. Furthermore, no previous studies have assessed the combined effect of NK, attitudes, perceptions, and challenges (APC) on dietary goals and intakes of rugby players. Therefore, this study aimed to assess the NK, dietary habits, and determinants of food choices of semi-professional rugby athletes in Scotland and explore their interrelationships.

## 2. Methods

### 2.1. Study design and participants

This cross-sectional study was carried out in April 2020 in Scotland using a mixed-methods approach. Both quantitative and qualitative data were collected to explore individual experiences, practices, and beliefs [12]. Semi-professional male rugby athletes were recruited from one team of the Super6 Scottish Rugby Union League on a voluntary basis. The selected sample size was based on the number of available participants. Data collection took place during the COVID-19 pandemic; therefore, online delivery of the questionnaires and interviews was the only viable option. Research objectives and methods were conducted according to the guidelines laid down by the Declaration of Helsinki (2013) and ethical approval was granted by the Queen Margaret University (QMU) Ethics Committee in April 2020. Written consent was provided by all participants.

### 2.2. Recruitment and procedures

A total of 27 players were contacted to participate in the study, of which 24 agreed to take part (89%). Participants were provided with an information sheet, a consent form and an introductory video as an invitation to participate in the research study. Participants were free to withdraw from the study at any point without explanation. Subjects who agreed to participate received an e-mail link to the ‘Online Surveys’ on a weekly basis [13]. Anonymity and confidentiality of participants’ responses were ensured through the use of 4-digit username generated by an online tool.

Participants were asked to complete a series of online questionnaires of 10–20 minutes each (see *Data Collection*) over a 3-week period. All players who completed the questionnaires were included in the data analysis. A subgroup from the team were voluntarily recruited to participate in a semi-structured online interview via Skype of duration 20–30 minutes. Researchers used a standardized proforma to ensure participants were interviewed in a consistent manner.

### 2.3. Data collection (questionnaires and interview)

A questionnaire on demographics and APC (*n* = 22) (Appendix 1) was developed in agreement with previously published studies [4,5,14]. Weight and height were self-reported by participants. Special dietary needs/conditions included but were not limited to vegetarians, diabetes, coeliac disease, and allergies. Dietary intakes were assessed using the EPIC-Norfolk Food Frequency Questionnaire (FFQ) (*n* = 22) that lists UK-specific food items [15]. A modified version of the Nutrition for Sports Knowledge Questionnaire (NSKQ) (*n* = 20) [14, 22] was used to assess participants’ nutritional knowledge (Appendix 2). All the aforementioned questionnaires were delivered using online surveys.

Additionally, individual interviews took place and the process was conducted virtually via Skype (*n* = 16). An interviewer guide was used to further explore participants attitudes, knowledge, and perceptions around diet (Appendix 3). More information and samples of the questionnaires and discussion proforma used can be found in supplementary material.

### 2.4. Data management

All data collected using Online Surveys [13] were exported to Excel, coded and transferred to Statistical Package for the Social Sciences (SPSS) for analysis. Interviews were recorded, later transcribed and coded. Data were stored in compliance with the general data protection regulation (GDPR) legislation.

### 2.5. Data analysis

The FFQs were analyzed using the FFQ EPIC Tool for Analysis (FETA) software [16]. The nutrient intakes of the group were compared to international sports nutrition guidelines for carbohydrate (5–10 g/kg body weight) and protein (1.2–2.0 g/kg body weight), [I; 17,18], and the UK public health guidelines for fiber (30 g/day), total fat (<35%), SFA (<11%), and alcohol (<14 units/week) [19,[20]]. Macronutrient intakes relative to body weight were calculated using self-reported body weight. A one-sample *t*-test or WiIcoxon signed-rank test was used to compare mean or median macronutrient intakes, respectively, to the relevant nutrition guidelines. Dietary intakes were compared to the lower end of the range for carbohydrate and protein. Body mass index (BMI) was calculated based on self-reported weight and height.

Statistical analyses were conducted in IBM SPSS ® version 23 (IBM Corp., Armonk, NY, USA). A preliminary analysis screening for missing values, outliers, and univariate normality of the data was conducted using Q-Q plots, skewness, and kurtosis statistics (−1,1). Distribution of continuous variables was assessed using formal tests of normality (Shapiro–Wilk). Continuous variables are presented as mean ± standard deviation (SD) and median (25th, 75th) as appropriate. Categorical variables are expressed as *n* (%). The level of significance was set at α = 0.05.

The sample was split into two groups (Good vs Poor knowledge) based on the median score to further investigate differences and relationships relative to nutritional knowledge. Mann–Whitney *U* and independent-sample *t*-tests were used to analyze differences in nutrient and food group intakes based on level of NK (Good, Poor) using a median split. Similarly, differences in NK scores and previous nutrition or health qualification (yes or no, based on self-report of course or degree completed), previous professional dietary input (yes or no) and performance enhancing supplement (PES) use (yes or no) were assessed using an independent-samples *t*-test/Mann–Whitney *U* test. Differences in knowledge scores based on highest level of education (GSCE’s, A-levels, undergraduate or postgraduate degree), and familiarity with the Eatwell Guide [yes, no or unsure) were assessed using ANOVA/Kruskal–Wallis. Where ANOVA results were significant, a Tukey post hoc analysis was conducted to determine which groups differed. Relationships between NK scores with dietary intakes and demographic factors were examined using Spearman’s Rank-Order correlations.

Triangulation was carried out by collecting data via questionnaires and interviews to increase the robustness of qualitative data. Thematic analysis was used to explore key ideas from the ‘APC’ questionnaire and Skype interviews. Researchers manually extracted themes by identifying common topics and repeating patterns from the qualitative questionnaire results and interview transcripts. Consensus regarding themes was achieved through discussion. Quotes were extracted to represent the themes identified, with selected verbatim quotes presented in the results. Methods are underpinned by those described in the qualitative research study conducted by Stokes et al. [5].

## 3. Results

### 3.1. Participant characteristics

Twenty-four male semiprofessional rugby players agreed to participate in the study. Response rate was 92% (*n* = 22) for the ‘Demographics & APCs’ and ‘FFQ’, and 83% (*n* = 20) for the ‘NKSQ’. A total of 16 (67%) players took part in individual Skype interviews. All participants were white, aged 20-34 with a mean± SD BMI of 27.5 ± 2.4 kg·m^−2^. Median (IQR) years of experience was 14.5 (12.0, 17.5) years. Participants classified their habitual physical activity as active (~1–3 h/day, 77%, *n* = 17), vigorous (>4–5 h/day, 18%, *n* = 4), or moderate (~1 h/day, 4.5%, *n* = 1). The median (IQR) hours of training per week were 7.5 (6.5, 9.0) hours (Table 1).

**Table 1.** Participant characteristics

*N* = 21	Mean ± SD/Median (IQR)	
Age (years)^a^	23.0 (21.5, 27.5)	
Height (m)^b^	1.87 ± 0.10	
Weight (kg)^b^	96.6 ± 10.8	
BMI (kg·m^−2^)^b^	27.5 ± 2.4	
Training per week^a^(h)	7.5 (6.5, 9.0)	
Game experience^a, c^ (years)	14.5 (12.0, 17.5)	
	*n*	%
***Ethnicity***		
White/Caucasian	22	100
***Position Played***		
Back	11	50
Forward	11	50
***Level of Education***		
GCSE	2	9
A-levels	8	36
Undergraduate degree	8	46
Postgraduate degree	2	9
Health-/Nutrition-related qualifications	3	14
Special dietary needs/conditions	1	5
Previous dietary/nutrition consultation	8	36
***Employment Status***		
Employed full-time	12	55
Student	7	32
Unemployed	2	9
Other	1	5
Dependents	2	9

^a^Expressed as median (25th,75th)   .

^b^Based on self-reported data.

^c^*n* = 20.

BMI, body mass index; SD, standard deviation; GCSE, General Certificate of Secondary Education.

All players had GSCEs as a baseline education qualification. Of those who had health-/nutrition-related qualifications, one player had a National Vocational Qualification (NVQ) in personal training and two had undergraduate degrees in sports and exercise science. It was reported that 36% of the respondents had previously consulted a registered dietitian (RD) or nutritionist. Of those who never had a consultation, five stated that they did not know how to access a consultation with a dietitian/nutritionist, four believed their own knowledge of nutrition was adequate, one did not specify a reason, and four responded as ‘other’ as the reason for no consultation. Two of the four participants responding ‘other’ as a reason stated: “*I do not really trust nutritionists. It seems to me they often contradict each other, and everyone seems to be claiming they are right* or *I never had the opportunity”*.

### 3.2. Dietary practices and beliefs

All players perceived healthy eating to be important, when asked about the benefits of healthy eating. When asked specifically about how nutrition is important for athletes, 91% of participants quoted improvements in performance, recovery, and training, and 96% understood that there are specific dietary recommendations for athletes. Some players believed PES are necessary for athletes (36%) and 68% reported using both PES and nutritional supplements (omega-3 fish oils, vitamins, probiotics). Table 2 summarizes athletes’ dietary practices and beliefs.

**Table 2.** Descriptive statistics of participants’ dietary practices and beliefs from the demographics and APC questionnaires

Dietary practices	*N*	Frequency
Ability to cook	22	100%
***Cooking and Shopping responsibly***		
Self	14	67%
Shared	2	9.5%
Partner	3	14%
Parents	2	9.5%
PES Use	15	68%
Nutritional Supplement Use	15	68%
***Understanding and Beliefs***		
Dietary Recommendations for Athletes	21	96%
Familiar with the Eatwell Guide	5	23%
Importance of Healthy Diet for Athletes	22	100%
Believe they follow a Healthy diet	21	96%
Diet affects performance	20	91%
PES necessary for athletes	8	36%

APC, Attitudes, perceptions, and challenges; PES, performance enhancing supplements.

### 3.3. Nutritional knowledge

Overall NSKQ scores were low, with an average of 44 items answered correctly out of the 82 questions. The score ranged between 34.2% and 79.3%, with a mean ± SD of 53.7 ± 11.9%. Table 3 shows the NK scores for each category and between-group comparisons. The ‘Good’ NK group scored significantly higher in the weight management (*p* = 0.014), macronutrients (*p* < 0.001), micronutrients (*p* = 0.001) and sports nutrition sections (*p* < 0.001).

**Table 3.** Mean nutritional knowledge results

Category (items)	All (*n* = 20)		Good NK (*n* = 10)		Poor NK (n = 10)		P-value
	Score	%	Score	%	Score	%	
Weight Management (9)	6.1 ± 17.4	67.8 ± 16.9	6.9 ± 1.2	76.7 ± 13.3	5.3 ± 1.4	58.9 ± 15.8	**0.014**
Macronutrients (29)	17.4 ± 3.9	59.9 ± 13.7	20.2 ± 2.3	69.8 ± 7.8	14.5 ± 3.2	50.1 ± 10.9	**<0.001**
Micronutrients (13)	4.2 ± 2.7	32.3 ± 20.5	6.0 ± 2.1	46.2 ± 15.8	2.4 ± 1.9	14.6 ± 15.8	**0.001**
Sports Nutrition (12)	6.7 ± 2.0	55.8 ± 16.4	8.1 ± 1.5	67.5 ± 12.1	5.3 ± 1.3	44.1 ± 11.1	**<0.001**
Supplements (12)	5.2 ± 2.2	42.9 ± 18.0	6.0 ± 1.6	50.0 ± 13.6	4.3 ± 2.4	35.8 ± 19.7	0.078
Alcohol (7)	4.6 ± 1.3	65.0 ± 18.8	4.9 ± 1.1	70.0 ± 15.7	4.2 ± 1.5	60.0 ± 21.1	0.245
Total (82)	44.1 ± 9.8	53.7 ± 11.9	52.1 ± 5.2	63.5 ± 6.4	36.0 ± 5.5	43.9 ± 6.7	**<0.001**

Bold values indicate P<0.05. Values are represented as mean ± standard deviation. NK,nutritional knowledge.

Neither age (*p* = 0.420) nor years game experience (*p* = 0.386) was significantly associated with NK scores. Relationships of all other participant characteristics with NK scores are displayed in Table 4. No associations were found between educational level and NK scores. Total NK% was not significantly associated with prior dietetic consultation; however, subjects who had previously had a consultation with a dietitian or nutritionist (*n* = 7) scored significantly higher in the Macronutrients (*p* = 0.025) and Sports Nutrition (*p* = 0.026) subsections. Participants with health- or nutrition-related qualifications scored higher overall in the NSKQ and (*p* = 0.036) and the sports nutrition sections (*p* = 0.030). Subjects that were familiar with the Eatwell Guide (*n* = 4) scored significantly higher in the NSKQ overall (*p* = 0.040) and the micronutrients section (*p* = 0.044).

**Table 4.** Mean nutrition knowledge scores as percentages according to participant characteristics

	Subgroup	Total NK	WM	Macro	Micro	Sports	Supplements	Alcohol
Education Level	*GSCE*	48.2 ± 14.6	50.0 ± 23.6	48.3 ± 24.4	34.6 ± 16.3	54.2 ± 29.5	37.5 ± 16.7	78.6 ± 10.1
	*A-levels*	55.0 ± 10.7	68.1 ± 12.5	60.1 ± 12.5	30.8 ± 18.8	57.3 ± 15.7	51.0 ± 16.3	66.1 ± 18.6
	*Undergraduate*	56.5 ± 13.6	73.6 ± 19.7	65.2 ± 11.2	37.5 ± 23.8	58.3 ± 16.6	37.5 ± 19.9	64.3 ± 18.7
	*Postgraduate*	42.7 ± 1.7	61.2 ± 7.0	50.0 ± 17.1	15.4 ± 21.8	41.6 ± 11.8	37.5 ± 17.7	50.0 ± 30.3
Dietary Input	*Yes*	59.7 ± 9.6	71.5 ± 15.5	**70.0 ± 9.7**^a^	34.1 ± 15.3	**66.6 ± 15.9**^a^	45.2 ± 15.1	67.3 ± 18.0
	*No*	50.5 ± 12.1	65.8 ± 17.8	55.1 ± 13.2	31.4 ± 23.4	50.0 ± 14.0	41.7 ± 19.8	63.7 ± 20.0
Health Qualification	*Yes*	**70.1 ± 12.9**^a^	88.9 ± 15.7	74.2 ± 17.0	50.0 ± 16.3	**79.2 ± 5.9**^a^	54.2 ± 5.9	78.6 ± 10.1
	*No*	51.9 ± 10.7	65.4 ± 15.7	58.4 ± 12.9	30.4 ± 20.3	53.2 ± 15.2	41.7 ± 18.5	63.5 ± 19.1
FAmiliar with Eatwell Guide	*Yes*	**66.8 ± 8.7**^a^	83.4 ± 19.2	71.8 ± 11.3	**53.9 ± 18.8**^a^	70.8 ± 15.9	52.1 ± 4.2	67.9 ± 27.0
	*No*	50.3 ± 9.3	63.5 ± 14.7	57.5 ± 12.8	25.8 ± 15.3	52.4 ± 15.5	39.9 ± 17.3	64.3 ± 18.4
PES Use	*Yes*	54.0 ± 13.2	69.2 ± 16.5	61.4 ± 16.5	31.4 ± 16.2	57.0 ± 18.6	39.1 ± 18.7	67.0 ± 18.8
	*No*	53.1 ± 10.0	65.1 ± 18.6	57.2 ± 5.8	34.1 ± 28.4	53.5 ± 12.6	50. ± 15.2	61.2 ± 19.7

^a^Denotes *p* < 0.05, values are mean ± standard deviation, *n* = 20.

^b^PES, performance enhancing supplement; NK, nutritional knowledge; WM, weight management; Macro, micronutrients; Micro, micronutrients.

### 3.4. Dietary intakes versus Guidelines

Estimated intakes from food sources were significantly lower than the recommended amounts for carbohydrate and fibre (all *p* ≤ 0.001). Only one player met the lower end of the ACSM [18) recommended carbohydrate range. The majority (89%) of players were not meeting carbohydrate recommendations and consumed significantly less (*p* < 0.001) than the recommended amounts for their activity level. Mean protein intakes fell within the recommended range for athletes [18]. The majority (58%) of participants consumed between 1.2 and 2.0 g/kg/day protein; however, 32% did not meet the lower end of the range and 11% exceeded the upper range. Total fat and SFA intakes exceeded the recommended amounts, but differences were only significant for SFA (*p* < 0.001). Table 5 summarizes the athletes’ estimated average intakes for energy, protein, carbohydrate, fibre, fat, and alcohol.

**Table 5.** Daily energy and macronutrient intakes of participants (*n* = 22) from food sources versus sports nutrition guidelines

Nutrient	Absolute daily intake	Relative Intake (per kg BW)	Recommendation	Intake vs Guidelines	*p*-Value
Energy (kcal)	2472 (1767, 3374)	26.3 ± 9.2	-	-	-
Protein (g)	139.8 ± 51.1	1.4 ± 0.4^a^	*1.2–2.0 g/kg BW*	Within	0.148
Carbohydrate (g)	266 (199, 341)	3 (2, 3)^a^	*5–10 g/kg BW*	Below	**<0.001**
Fibre (g)	21.7 ± 10.1	-	*30 g/day*	Below	**0.001**
Total fat (g)	97.0 (68.3, 150.3)	36.2 ± 3.7^b^	*<35% TEI*	Above	0.149
Saturated fat (g)	38.7 ± 15.8	12.8 ± 1.9^b^	*<11% TEI*	Above	**<0.001**
Alcohol (units/week)	6.3 (2.3, 10.6)	-	*<14 units/week*	Below	**<0.001**

^a^*n* = 19; values are mean ± standard deviation/Median (25th, 75th). ^b^Expressed as %TEI; BW, body weight; TEI, total energy intake. *Average protein and carbohydrate intakes compared to* ACSM [18] *guidelines, mean fibre intake compared to SACN [42] guidelines, mean total fat & saturated fat intakes compared to SACN [43] guidelines, Average weekly alcohol intake in units compared to the* Department of Health [19] *guidelines.* Bold values indicate P<0.05.

### 3.5. Nutritional knowledge and dietary intakes

Those with good NK consumed vegetables, breakfast cereals, wholemeal pasta, sweets and snacks, bacon, chips, and pizza more frequently than the poor NK group. Table 6 displays the total estimated average intake for each food group. The ‘Good’ NK group had significantly higher intakes of all macronutrients, meat, and dairy products (all *p* < 0.05). Spearman’s correlations showed a significant positive association between NK and carbohydrate intake (*r* = 0.652, *p* = 0.015) and protein intake (*r* = 0.649, *p* = 0.015).

**Table 6.** Athletes average daily food consumption and between-group comparisons according to level of nutritional knowledge

Food group	All (*n* = 20)	Good NK (*n* = 10)	Poor NK (*n* = 10)	*p*-Value
Energy (kcal)	2580.9 ± 944.9	3135.2 ± 1005.9	2026.5 ± 436.2	**0.015**
Carbohydrate (g)	295.6 ± 124.7	327.7 ± 106.2	228.7 ± 56.7	**0.019**
Protein (g)	134.5 ± 48.3	160.5 ± 53.0	108.5 ± 24.7	**0.023**
Fat total (g)	106.6 ± 46.7	134.8 ± 49.3	78.4 ± 20.1	**0.007**
SFA total (g)	37.3 ± 14.5	45.8 ± 13.7	28.8 ± 9.9	**0.009**
Iron (mg)	15.1 ± 6.1	18.3 ± 7.0	11.8 ± 2.6	**0.023**
Fiber (g)	21.7 ± 10.1	26.5 ± 12.2	16.8 ± 3.4	0.089
Nonalcoholic beverages (g)	628.6 ± 445.5	713.1 ± 542.0	544.1 ± 330.7	0.450
Cereals & cereal products (g)	300 (193, 437)	400.1 ± 235.3	275.7 ± 108.7	0.247
Meat & meat products (g)	233.2 ± 104.9	280.3 ± 111.6	186.1 ± 76.3	**0.043**
Dairy & dairy products (g)	592.1 ± 282.7	717.5 ± 316.2	466.7 ± 183.8	**0.043**
Fish & fish products (g)^a^	30 (9, 52)	29.5 (11, 65)	29 (3, 57)	0.796
Eggs & egg dishes (g)	40 (11, 50)	40.6 ± 35.8	32.4 ± 17.3	0.631
Fruit (g)^a^	309 (188, 423)	329 (197, 430)	249 (125, 426)	0.436
Vegetables (g)	338.8 ± 163.4	410.9 ± 195.0	266.7 ± 82.1	0.075
Potatoes (g)	66.9 ± 43.2	79.0 ± 48.3	54.7 ± 35.8	0.280
Fats & oils (g)^a^	12 (7, 14)	13 (10, 29)	9.5 (2.5, 13)	0.063
Nuts & seeds (g)^a^	17 (5, 36)	22 (11, 53)	8 (2, 25.5)	0.089
Sugars & snacks (g)	36.2 ± 22.2	35.6 ± 18.7	36.8 ± 26.0	0.971
Soups & sauces (g)	81.6 ± 65.7	103.7 ± 68.7	59.5 ± 57.4	0.123
Alcoholic beverages (g)^a^	111 (30, 149)	138 (82.5, 193.5)	40 (21.5, 129)	0.052

Bold values indicate *p* < 0.05; values are mean ± standard deviation. ^a^Expressed as median (25th, 75th). NK, nutritional knowledge; SFA, saturated fatty acids.

### 3.6. Perception of diet and performance identified during interviews

Of the 24 total respondents, 16 (67%) agreed to participate in Skype interviews. Athletes reported healthy eating can improve performance and recovery, and positively impacts immune function, health, and well-being. Free time for planning meals, preparation, and bulk cooking was perceived to be the largest contributor to healthy eating habits when asked about factors that enable or conversely create barriers to healthy eating. Additionally, building healthy habits through discipline and peer/family influence, as well as awareness of healthy foods were cited as the most common factors in maintaining a healthy diet. In relation to nutrient goals, participants struggled to meet, more than half reported difficulties in achieving the recommended protein intake. Foods/food groups that were perceived as healthy included fruit, vegetables, wholegrains with a mixture of carbohydrate and protein sources as well as steak, chicken, apples, banana, broccoli, pasta, rice, potatoes, oats, eggs, yogurt and nuts.

Tables 7 and 8

**Table 7.** Attitudes, perceptions and challenges of players surrounding diet, performance, and PES identified in interviews

Skype interview topics	Attitudes, perceptions & challenges	% of population in agreement (*n* = 16)
*Perceptions of a healthy diet*	- A good balance of all food groups, without relying too heavily on one source - Quality, natural ingredient sources - Helps improve athletic performance - Contributes to mood and mental health - Enhances recovery, sleep, digestion and immune function - Contributes to feeling ‘healthier’ and ‘looking better”	94% 31% 31% 63% 25% <20%*
*Perceptions of an unhealthy diet*	- High sugar content - Heavily processed - Snack foods including; chocolate, crisps & sweets - Take-away foods including; pizza, fried foods, fish & chips - Sugary drinks - Ready meals - Overeating/relying excessively on one food group - Foods that make you feel ‘sluggish’, ‘lethargic’ or ‘bloated’	50% 50% 88% 63% 25% 20% 63% 31%
*Impact of diet on performance*	- Diet significantly impacts performance - Quality of meals contributes to performance - Quality and timing of meals influences performance; - Fueling several hours before a game and immediately after improves performance - Not eating enough or too close to a game negatively impacts performance - Timing of meals does not impact performance - Meeting carbohydrate and protein requirements is important - Hydration is an important factor to enhance performance	100% 44% 56% 25% 25% <20%* 69% 25% <20%*
*Thoughts on PES*	- PES are beneficial, convenient or practical - PES give you an ‘edge’ to improve performance or strength - PES are non-essential - A well-balanced diet should be able to provide all nutritional requirements	46% 23% <20%* 50%
*Specific foods, drinks or products used to aid training and recovery*	- Whey protein - Caffeine - Creatine - BCAAs, glucose, energy gels, beetroot shots or isotonic sports drinks	69% 44% 38% 25%
*Dietary goals*	- Weight gain - Maintain current weight - Reduce fat mass and increase muscle mass - Maintaining a well-balanced diet - Avoid foods high in sugar and processed foods - Increase dietary protein & carbohydrates - Increase healthy snacks/fruit to achieve a healthy balanced lifestyle - Do no track dietary intake therefore not aware of nutrient intakes versus goals	31% 44% 63% 38% <20%* 25% <20%* <20%*
*Time of year when nutrition is a priority*	- All year-round - Pre-season - Less focused during holidays - Time of year can be a challenge in achieving dietary goals	44% 44% 25% <20%*
*Determinants of dietary choices* *Barriers to meeting dietary goals*	- Planning and preparation make healthy eating easier - Positive habits, influences and good NK - Enjoyable and easily prepared foods - Looking and feeling good - Lack of time for meal planning, preparation and cooking - Lack of motivation, discipline, tiredness - Taste preferences, menu fatigue, lack of knowledge - External influences and dietary requirements of other members of the household - Training schedule impacting on time for preparation - Stress, sleep, alcohol, social life - Cost	69% 20% 31% 25% 69% 25% <20%* 25% 31% <20%* 25%
*Impact of coronavirus*	- Easier to meet dietary goals than before with more time for planning and preparation - Adjusting diet to maintain body composition with restricted access to weights as a significant challenge - Reduced motivation - Changes in appetite - Reduced food availability - Difficulty establishing routine and planning ahead - Reduced disposable income - Not tracking dietary intake during lockdown - Struggling to meet protein goals - Struggling to adjust volume of food for new energy requirements - Reduced muscles mass, fitness and strength likely after the pandemic - Training at home is adequate to maintain body composition with minimal effects on performance	<20%* 50% 25% <20%* 31% 38% <20% 38% 31% <20%* 94% <20%*

* Where the number of participants was less than 3, figures were reported as <20%

**Table 8.** Themes and quotes extracted from individual interviews

	Themes identified	Noteworthy responses
Perception of a healthy diet	Balance, variety enhanced performance, recovery, mental health, well-being, and energy levels	*‘Making sure you get enough protein, vegetables, fruits, vitamins, not too many processed foods or red meat. It’s important for quality of life, I feel more energetic when eat healthily and I can do more’.*
		*‘A balanced diet, hitting all the food groups but not eating one thing in excess. It helps with everyday life for work and sport, mentally and physically you can feel the benefits’.*
Perception of an unhealthy diet	High sugary content, heavy processing, food groups in excess	*‘Overindulgent, high sugar processed, too much food, high calorie foods, foods easy to snack on’*
		*‘Overeating or undereating, both can be unhealthy in different ways, consuming one food group too much like a certain carb, or binge eating on unhealthy foods high in sugar, saturated fat and processed foods’.*
Influence of diet on performance	Energy to sustain exercise Aids recovery Enhanced athletic performance Improved mental focus	*‘The times where I fuel accordingly, I can definitely see the peaks in my performance. If I get my nutrition right prior to training I can last the duration, whereas if I don’t, I really struggle’.*
		*‘If I eat the right amount of food, I feel like I have more energy, If I don’t eat properly, I feel light-headed, low in energy during a game, or it will impact my ability to train’.*
Aspects of nutrition influencing performance	Carbohydrate intake Quality of meals Meal timing Volume of food Hydration	*‘Release of energy*, you *don’t want to go half-way through a game to see that you have no energy left. Goal would be to maintain energy throughout; mainly from carbohydrate’.*
		*‘Carbs as the main source of energy – the right type of carbs too. Hydration so you can function properly. I don’t like to eat too much the morning of the game; I eat little before the game and slightly more for dinner the night before’.*
Nutritional strategies	Supplements Pre-game/ Post-exercise meal Hydration Carb loading Protein intake	*‘I try to come off caffeine a day or two before a game; it works better in terms of being stimulated for the game’.*
		*‘II will take beetroot juice an hour and a half before training for the greatest impact according to the advice’*
		*‘The night before a game, I make sure I have a decent meal with decent amounts of protein and carbs’*
		*‘I try to drink as much water as possible, I am not very good at it, but I try’*
Impact of meal timing	Gastrointestinal discomfort Fatigue Prevention Aids Recovery	*‘I eat a lot of food after training to refuel, before I try not to eat too much to avoid feeling sick’.*
		*I think it’s especially important before games and training. If I don’t eat enough or eat too much, I will struggle at training. Important to keep yourself fueled up throughout the day and after games too for recovery’*
		*‘Not too bothered about timing; I eat as I normally would; don’t change timing of meals even during a game unless I have to’*
Determinants of dietary choices	Time, planning, preparation Work/family/university commitments Convenience, motivation, habits Preferences, cost, availability	*‘Biggest thing that affects me is time, I am generally very busy which means I can be lazy with meal prep’*
		*‘Time management for meal prep, personal motivation, availability of correct foods and money in relation to supplements which can be expensive especially as I am a student’*
		*‘Convenience, if I am short on time due to work or a long week and I want something I can put in the oven’*
Dietary goals	Increase muscle mass, strength, weight, protein Reduce fat, processed foods Healthy/balanced diet Not sure/None	*‘Remain the same weight, cut body fat, build muscle, I try to reduce carbs and maintain fat intake’*
		*‘Making sure I have enough protein, not too many processed foods to keep muscle mass and lose a bit of fat’*
		*‘Maintain a well-balanced diet and stay away from snacking. I try to have three meals and Increase muscle mass’*
		*‘I don’t really have specific goals I generally just try and eat quite healthy’*
Barriers to dietary goals	Meal planning, preparation, cooking, personal discipline, motivation, special dietary requirement, finances	‘*Probably time and planning, so planning ahead in terms of when I am going to do a shop, its usually at the weekends so I need to plan ahead what I am going to eat for lunch, snacks and presession so that I can prepare them accordingly throughout the week’*
		*‘I have been doing alright actually from a nutrition perspective because I have more time to put into it and my evening are freed up to do some more cooking’.*
		*‘Having the motivation to cook for 45 minutes when life happens when I could easily just snack on a bowl of cereal’*
		*‘Money. My weight is 108 kg, so I need 2 grams of protein and that costs a lot of money’*
Challenges of coronavirus	Maintaining body composition Training limitations Time/planning, food availability Easier/Not sure/None	*‘I have been doing alright from a nutrition perspective because I have more time to put into it and my evenings are freed up to do some more cooking. Training has been more difficult because I don’t have the equipment’.*
		*‘I don’t really have goals as such around what I am trying to do, I am aware of what I’m eating but I wouldn’t say I am eating any better or worse than I was before’.*
Impact of coronavirus	Reduced muscle mass, fitness, strength, motivation Minimal/Not sure/None	*‘Muscle mass and body weight may drop due to less training; maintaining weight is quite tough. I try to do as much training as I can but as we cannot train with other players, I may not push myself as much and have less motivation’*. *‘Corona virus will have a big impact on reconditioning side if it stays any longer. I may lose muscle mass and it may change my body composition’*. *‘I don’t think it will have a huge impact because I am still training at a decent level and we would usually be having a bit of a rest at this time of year anyway’.*

summarize the players’ responses to interview questions, themes extracted, and supporting quotes.

## 4. Discussion

The mean NK score of semiprofessional players in this study was 53.4%. The observed score in the present study is significantly lower compared to other studies on elite rugby athletes (73% [4] and 61.3% [21]). However, this study used an adapted NSKQ consisting of 39 questions [14] whereas the other two studies used a questionnaire with different scoring systems (a 90-point and 72-point GNKQ) [4,21,22]. Disparity in results may be due to different assessment tools used. Moreover, semi-professional players may have limited opportunity to access nutritional resources and advice, therefore lower NK in comparison to elite athletes may be expected [23, 24].

The differences in the scores of the good NK and poor NK groups were significant for all sections excluding supplements and alcohol. Discrepancies between studies make it difficult to compare scores as different tools, nutrition subsections, cutoffs, and sample characteristics have been used [4,21,25]. However, a common theme between the current findings and other studies has emerged [25,26] and reveals that athletes’ knowledge of dietary guidelines and supplementation is poor.

In a study with Australian football players, it was noted that most players were aware of the alcohol recommendations; however, only a few were able to identify a unit of alcohol [27]. Given the drinking culture in the sports industry [28] it is surprising to have a gap in knowledge in this area [29,30, 31]. However, in contrast to previous findings, the average alcohol intake of the rugby players in this study was significantly lower than the maximum allowance of 14 units per week (median (IQR) 6.3 (2.3, 10.6), *p* < 0.001). In addition to general health benefits, this is a noteworthy finding as most players were striving to maximize anabolism, and research has shown that muscle protein synthesis can be suppressed if alcohol is consumed, even in the presence of adequate protein intake [32].

Contrary to the hypothesis, neither increased age, years game experience, level of education obtained, nor employment status were associated with improved NK or DIs. This study did not find significant differences in NK between all levels of education. This may be due to participants’ level of education [100% had GSCEs). These findings are similar to those of Spronk et al. [33] however are in contrast to the Trakman et al. [25] study which found NK to be influenced by education. Respondents that were familiar with the Eatwell Guide, scored significantly higher in the NSKQ overall and the micronutrients sections. Similar to the findings of Andrews and Istiopoulos [34], participants with health-related qualifications scored higher overall in the NSKQ and the sports nutrition sections. Total NK% was not significantly correlated with dietary input; however, subjects who had previously had a consultation with a dietitian or nutritionist scored significantly higher in the Macronutrients and Sports Nutrition subsections, which may be related to the information received during the consultation. Participants in this study who have not had a prior consultation with a dietitian/nutritionist reported a range of reasons including problems accessing a dietitian/nutritionist, belief around the adequacy of their own NK and lack of trust. It is common for semiprofessional athletes to have trouble accessing RDs due to limited resources and time [35]. Athletes’ supplements use is frequently directed by family, friends, teammates, coaches, athletic trainers, and the media instead of RDs/nutritionists [18,36].

The current results showed the players’ mean energy intake from food sources was 26.3 ± 9.2 kcal/kg body weight, which may be insufficient to meet energy demands. However, fluctuation of dietary intakes throughout seasons according to training demands were not accounted for in the dietary analysis. Average carbohydrate intake (3 g/kg/day) met ACSM sports nutrition guidelines for skill-based activities (3–10 g/kg/day). However, intakes for 89.5% of the group were below the recommendation of 5 g/kg/day for athletes performing moderate-intensity daily exercise. Notably, carbohydrate intake may have been underestimated as supplement use was not accounted for in the calculation of carbohydrate intake. These results were consistent with findings from previous studies of rugby athletes where carbohydrate intakes have frequently been shown as inadequate [4,37,38]. Similar to the findings of 39, the current mean fibre intake (21.7 ± 10.1 g/day) was less than the recommended amount (30 g/day).

Total fat %TEI did not differ significantly from the guidelines, and mean daily protein intake (1.4 ± 0.4 g/kg/day) was in line with ACSM [18) sports nutrition guidelines, in contrast to past research where protein intakes of rugby players exceeded recommendations [37,38]. However, 31.6% of the study population did not meet the lower range of the recommended amounts (<1.2 g/kg/day) from food sources. Interestingly, 68% participants reported taking whey protein supplements; therefore, it is possible that players with intakes below the recommended amount were meeting their protein requirements through a combination of food sources and supplements. Although some results were inconsistent with previous findings [38], the overall macronutrient and energy intakes were in agreement with the findings from a recent systematic review in which dietary intakes of semiprofessional team sports athletes were found to meet or exceed the recommendations for protein and/or fat, but were inadequate for carbohydrate and energy [6].

Overall, the good NK group had higher energy and macronutrient intakes than the poor NK group, suggesting the good NK group would be more likely to meet their energy and protein requirements from food sources. However, the present study revealed that most participants had carbohydrate intakes below the lower range of ACSM guidelines, and particularly those from the poor NK group (80% versus 56% for good NK group), which is consistent with previous findings [1, 2, 59-60]. Higher NK has been positively associated with consumption of fruit, vegetables, and carbohydrate-rich foods [3, 4]. However, there are controversial beliefs around carbohydrate in sports nutrition as over the years there has been a downward trend in carbohydrate intakes among athletes including rugby players, which may be influenced by motives to reduce body fat and optimize training adaptations [59]. This may be caused by how the media portrays nutritional information in relation to governmental dietary guidelines [9]. Interestingly, a recent study found that adherence to nutritional guidance was seasonal among high-performance athletes and similar to the present study, dietary behaviours were underpinned by emotional barriers/motivation with training schedules limiting opportunities for food planning [40].

Results from a systematic review suggest weak but significant positive correlations between higher NK and DIs, and particularly intake of fruits and vegetables [33]. Participants in the current study reported that natural foods or a well-balanced diet should provide all requirements, which is in line with the British Dietetic Association (BDA) recommendation for sports [41, 42]. Stokes et al. [5] also reported adolescent rugby players to perceive similar foods as healthy. Foods reported as unhealthy by the participants in this study included sugary foods, takeaways, and sugary drinks, which was consistent with other studies [5,20,43]. Common limitations in studies include quality issues with only a few studies using validated instruments to assess NK and DIs [8,33,44].

Perception of healthy eating of participants was in line with the messages from the Eatwell Guide in this study. A study investigating dietary practices of elite athletes in Australia found similar results with participants describing healthy eating as achieving balance and having a variety of foods [20]. Participants of the current study emphasized the need for moderation, which agrees with previous findings [45]. A theme emerged from this study in which players described healthy eating as a perfect balance between reward (e.g. ‘cake’) and self-control (‘unhealthy when in excess’). This cultural norm of associating food high in sugar and fat as a treat was also noted among male college hockey athletes who reported the need for balance between self-sacrifice and indulgence [43]. This is explained by Connors and colleagues [46] as balancing strategies used by adults to make food choices where there are competing priorities of values such as health and taste.

The current study reported that 68% of players were taking supplements to enhance performance, which is line with other studies, with prevalence of supplements use ranging between 37% and 89% [46,47]. Attitudes toward PES use were variable; 23% of players felt PES gave them a competitive advantage, whereas 46% utilized them for convenience and 50% considered them non-essential. Similarly, in Potgieter et al. [38] and Bradley et al. [59], PES use was high and all players that used supplements took whey protein. Furthermore, 31% used caffeine prior to a game for and 25% utilised various other PES to aid with training and recovery. Participants used supplements to boost performance, to help achieve nutrient goals, optimize recovery, immune function, improve body composition and compensate for a poor nutrition, which is in accordance with other studies [5,36,47]. A systematic review has shown that elite athletes are more likely to use PES than semiprofessional athletes [48] and a recent study of rugby players suggested that the prevalence of ergogenic supplements use was greater among professional rugby athletes than amateur players [49]. However, it could also be argued that non-elite athletes are likely to use supplements to gain a competitive edge [50].

In this study, all participants reported to have cooking skills, which has been considered an enabler to healthy eating [43]. The majority of players (67%) reported being primarily responsible for the cooking and shopping in their household; however, some players stated that preferences of other members of the household influenced their dietary patterns. Studies have shown that accommodating family or friends’ food preferences can affect the athletes’ determination to maintain dietary goals [5,51,52]. Cost, convenience and availability were also influencing factors; however, contrary to previous findings [5,20], cost was not a primary determinant of food choice for most players in the current study. This is most often observed among those with limited finances such as low-income groups, students and adolescents [53,54, 55, 56].

Participants of this study also described decreased personal motivation/discipline as a challenge to maintain dietary goals. This is particularly evident during off-season among elite athletes, with some drastically changing their eating practices [20,43]. Similar to the findings of 20, many respondents considered lack of time and preparation to be the primary challenge toward healthy eating healthy and meeting dietary goals. Although the majority reported lack of time to prepare meals as a barrier, a few reported that COVID-19 lockdown has enabled them to have more time to prepare and cook meals. An Italian survey found similar results with participants having more aspiration to cook, which has led to higher consumption of homemade foods [57]. Moreover, a study of professional/semiprofessional rugby union players in New Zealand found the majority of players to have a reduced intake of packaged/convenience foods and greater fruit and vegetable consumption during the COVID-19 pandemic. Findings were similar to the present study, and players reported lack of motivation and limited access to training equipment as challenges during lockdown [58]. Concerns over the impact of reduced training and its impact on body composition were cited by a few players, who stated that it may have detrimental impact on future performance. Elite athletes may be provided with home-based exercise programs. and in some cases live video training sessions led by fitness trainers [59]; however in this study, none of the participants reported access to such opportunities.

### 4.1. Strengths and limitations of the study

To the researcher’s knowledge, this is the first study to address NK and APC around dietary goals and dietary practices of semiprofessional rugby players in Scotland. The researchers used both quantitative and qualitative methods, which enabled a more complete picture of the investigated research field [60]. Some of the tools used were validated, which enables comparison with other studies. However, the ‘Demographics & APC’ questionnaires were developed by the researchers, therefore, not standardized for use in this specific population, and the NSKQ was adapted, and therefore was not fully validated. The EPIC FFQ is a validated tool but not for athletic populations, and additionally, it may not provide an accurate measurement of fiber and alcohol intake. Moreover, FETA software does not account for supplement use in the calculation of macronutrient intakes. Intentional underreporting/overreporting as well as self-reported height and weights may have affected the accuracy of the results. Interviews were carried out individually and therefore the influence of peers, family, and coach was significantly reduced. Additionally, all participants were asked the same interview questions ensuring responses were not led by the researchers’ perspective. However, participants may have answered some of the questions to please the researchers and to meet perceived beliefs. Furthermore, researchers tried to minimize bias in retrieving themes during the analysis by triangulation of data. Additionally, the researchers were able to investigate the impact of COVID-19, as this is the first time since the second world war that athletes have had to interrupt competition [59]. The recommendations from this research (Table 9) could be potentially used by professionals working with athletes in the UK ensuring optimum nutrition despite limited resource availability [24].

**Table 9.** Recommendations

Athletes would benefit from increased nutrition education with specific attention to significance of adequate carbohydrate and energy intakes for performance and recovery.
Athletes should be made aware of sports nutrition guidelines and further emphasis should be put on the achievement of carbohydrate and energy goals alongside protein targets.
Further education around knowledge of supplement use is required and players should be educated that achievement of body composition goals should not come at the expense of carbohydrate and energy requirements to support recovery.
Specific nutritional strategies such as meal timing and consumption of appropriate pregame and recovery meals may be encouraged to enhance performance, recovery dietary practices of the players.
Provide educational tools around nutrition to athletes as well as athletes’ closed ones including coach, family, friends, partners and peers to create an enabling environment for healthy eating. Resources given could include but not limited to the following: Quick, healthy, low-cost recipe ideas Meal planning self-help guide Nutritional knowledge tailored to dietary goal of athletes Performance enhancing supplements and evidence. What and when is it needed?
Advise athletes on some of the limitations associated media’s nutrition advice and provide a list of government/evidence-based resources and contact details (if able) to increase knowledge or if further information is sought
Create a supportive environment within the team by for instance, developing a themed afternoon once every 3 weeks to share views and discuss some of the enablers/challenges around nutrition and performance
Have innovative ideas to maintain contact and/or train together during off-season or COVID-19 lockdown with peers and coach via means such as zoom video call

In conclusion, the current group of semiprofessional rugby players appeared to lack nutritional knowledge, and awareness of current sports nutrition guidelines. Moreover, they reported inadequate carbohydrate and fiber intakes from food sources. Players with good NK had significantly higher total energy, carbohydrate, and protein intakes. Despite average protein intakes meeting the recommended amounts, most players took whey protein supplements due to the perceived benefits of enhanced performance and body composition adaptations. The main challenge identified by players in achieving their dietary goals was lack of time for meal planning, preparation, and cooking. Thus, some players found it easier to meet their dietary goals during the COIVD-19 pandemic with more free time; however, restricted access to equipment was a significant challenge in meeting body composition goals during the same period.

## Supplementary Material

Supplemental Material

## Data Availability

The datasets used and/or analyzed during the current study are available from the corresponding author on reasonable request.

## Author’s Information

Sonam Hitendre, RD, MSc Dietetics and MPH, Masters of Public Health Nutrition.

Rebecca Jordan, RD, MSc Dietetics and BSc Nutrition.

Christos Theodorakopoulos, PhD, Lecturer in Nutrition, School of Health Sciences, Queen Margaret University.
