# Supplementary material for: Dietary Intakes, Knowledge, and Perceptions of Semi-professional Rugby Athletes in Scotland
Source: J Int Soc Sports Nutr. 2022 Mar 26;19(1):49–69. doi: 10.1080/15502783.2022.2036436 (PMC9116392; doi:10.1080/15502783.2022.2036436)
Supplement: Supplemental Material [file RSSN_A_2036436_SM2169.docx]

**Supplementary material**

**Table 1. A summary of questionnaires, surveys and interview techniques used in the current study.**

| 1. *Demographics and Attitudes, Perceptions, Challenges (APC) questionnaire*   The ‘Demographics and APC’ questionnaire was newly developed by the research team to capture participant characteristics as well as their APC around dietary goals using other studies as a guide (Alaunyte et al. 2015; Trakman et al. 2017; Stokes et al. 2018). The online questionnaire consisted of 36 questions, divided into two parts - “about you” and APC. Most questions were closed to ensure high participation rate and facilitate data analysis. |
| --- |
| 1. *Dietary assessment – Food Frequency Questionnaire (FFQ)*   The European Prospective Investigation into Cancer and Nutrition (EPIC)-Norfolk FFQ (Bingham et al. 2001) is a semi-quantitative tool designed to assess habitual DI over the course of the previous year and has been validated for use in the EPIC-Norfolk study population which reflects the DI of adults in the UK (Mulligan et al. 2014) and was chosen due to its availability of local food items. It was chosen to assess the players habitual DI, reduce reporting bias, maximise follow-up and reliability of data as it has shown reproducibility and relative validity for most nutrients (Bingham et al. 1997; McKeown et al. 2001). The EPIC-Norfolk FFQ has been extensively chosen as a method of choice and is widely used in UK studies (Bingham et al. 1997; Bingham et al. 2001; McKeown et al. 2011). |
| 1. *Nutritional knowledge*   NK was assessed using an adapted version of the Trakman et al. (2017) Nutrition for Sports Knowledge Questionnaire (NSKQ) consisting of 39 questions with six subsections; weight management, macronutrients, micronutrients, sport nutrition, supplementation and alcohol. The NSKQ was chosen as a rapid and easy tool for subjects to complete.  The tool was validated for use in the Australian population using Rasch analysis, having met all psychometric criteria for construct validity, internal reliability and test-retest reliability (Trakman et al. 2017). The questionnaire was adapted for use in our study population to reflect current UK dietary guidelines. |
| 1. *Skype Interviews*   Researchers carried out individual skype interviews to further explore players thoughts and approaches to dietary practices. Care was taken not to lead participant responses. Researchers probed participants to elaborate on ideas and thematic saturation was achieved.  Researchers used the same interview discussion guide. |

**Appendix 1: Demographic & APC Questionnaire**

**Part 1: About you**

| 1. Date of birth (DD/MM/YYYY) |
| --- |
| 1. Where do you currently live?   Postcode:  City:  Country: |
| 1. What is your race or ethnicity?   White/Caucasian  Black/African American  Asian/Pacific Islander  Hispanic/Latino  Other |
| 1. Who do you live with? |
| 1. Do you have any children under the age of 18 living with you? Or anyone that you care for?   Yes / No |
| 1. Have you ever had a consultation with a dietitian or a nutritionist?   Yes / No |
| 1. If not, why?   I don’t know how to access a consultation with a dietitian/nutritionist  I think I have a good knowledge of nutrition and don’t need it  Only elite athletes need a consultation with a dietitian/nutritionist  Other:  Specify other reasons/barriers: |
| 1. Employment   Employed full-time  Employed part-time  Student  Unemployed  Other  Please specify profession if currently employed: |
| 1. Highest level of education   Didn't finish high school  GCSE’s  A-levels  Undergraduate/Bachelor’s degree.  Postgraduate degree (PGDip)/ Master’s degree  Doctoral degree (PhD)  Please describe the name of the course: |
| 1. Health or nutrition related qualifications:   Yes / No  If yes, please specify: |
| 1. Number of years game experience: |
| 1. Position played:   Back / Forward |
| 1. What is your height?   Specify units: (ft and inches or metres or centimetres) |
| 1. What is your weight?   Specify units: (stone and pounds or kilograms) |
| 1. What is your dietary goal(s)? |
| 1. Do you have a special diet or dietary needs? (e.g., vegetarian, allergies)   Yes / No  If yes, please specify: |
| 1. Do you have any health conditions? (e.g., diabetes) |
| 1. Who does the shopping and cooking at home? |
| 1. Do you cook?   Yes / No  If not, for what reasons? (e.g., can’t cook) |
| 1. How would you classify your habitual physical activity?   Sedentary/ light  Moderate (e.g., 1h per day)  Active (e.g., 1-3h per day)  Vigorous (e.g., >4-5h per day)  How many times a week do you train? (e.g., 3 times a week)  How long do you train for? (e.g., 2 hours)  What type of exercise do you do? (e.g., weightlifting for half an hour and running for one hour) |
| 1. Are you taking any medication that may influence your performance?   Yes / No |
| 1. Are you currently taking any nutritional supplements?   Yes / No  If yes, state the name and the dose: |

| Part 2: Attitudes, Perceptions and Challenges |
| --- |
| 1. What do you think the benefits of healthy eating are? |
| 1. Do you think eating healthy is important for an athlete?   Yes/ No (In both cases, please specify why?) |
| 1. Do you think you have a healthy diet?   Yes/No |
| 1. What makes it easy for you to eat healthy? |
| 1. What are the challenges or main barriers you have to eating healthy? |
| 1. Is there a goal for a particular nutrient (e.g., protein, fat, carbohydrate, fibre, vitamin/mineral or daily amount of calories) that you struggle to meet and why? |
| 1. Do you think there is a relationship between what you eat and how you perform?   Yes/No |
| 1. What aspects of nutrition in particular, if any do you think, influence how you perform? Why? (Expand more on your answer to question 6 above) |
| 1. Do you think performance enhancing supplements are required for a rugby athlete? State why   Yes/ No (In both cases, specify why) |
| 1. Are there any specific foods, drinks, or products that you take to help you enhance your performance? Describe the products used: |
| 1. What are the main factors that influence what or how you eat? |
| 1. What are your main dietary goals as an athlete? |
| 1. What nutritional strategies if any do you apply to achieve your goals as an athlete? |
| 1. Do you think the dietary recommendation of the general population is different from the recommendation of rugby athletes?   Yes / No /Not sure |
| 1. Have you heard of the Eatwell guide before? Or seen the picture below? 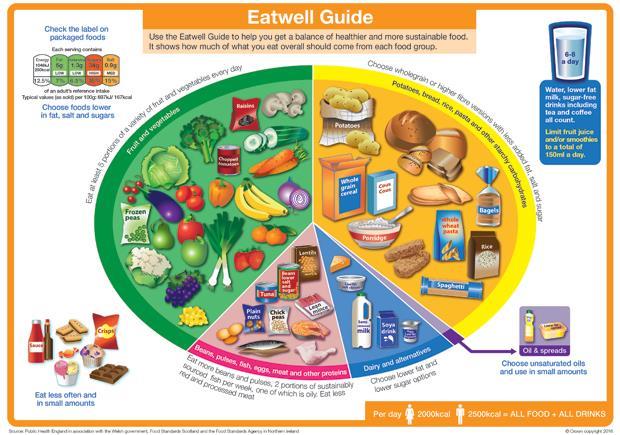   Yes / No / Not sure |

**Appendix 2: NSKQ**

**Q1.1 Which nutrient do you think has the most energy (calories) per 100 grams?**

- Carbohydrate
- Protein
- Fat
- Not sure

**Q1.2 Do you think the diet changes below are good ways to lose weight?**

|  | Yes | No | Not Sure |
| --- | --- | --- | --- |
| 1. Swapping carbohydrates/energy dense foods for low-energy foods like vegetables (Yes) |  |  |  |
| 2. Eating margarine instead of butter (No) |  |  |  |
| 3. Eating protein bars and shakes instead of yogurts, muesli/granola bars and fruits (No) |  |  |  |
| 4. Choosing lower glycemic index (GI) carbohydrates to help regulate appetite (Yes) |  |  |  |

**Q1.3 If they want to lose weight, athletes should:**

- eat less than 50 grams of carbohydrate per day
- eat less than 20 grams of fat per day
- eat less calories than your body needs
- Not sure

**Q1.4 To ensure they meet their energy (calorie) requirements, all athletes should:**

- plan their diet based on their age, gender, body size, sport and training program
- eat based on their natural hunger and fullness signals
- eat at least 2000 calories per day
- eat more foods that have lots of carbohydrate
- Not sure

**Q1.5 Which is a better recovery meal option for an athlete who wants to put on muscle?**

- A 'mass gainer' protein shake and 3 - 4 scrambled eggs
- Pasta with lean beef and vegetable sauce, plus a dessert of fruit, yoghurt and nuts
- A large piece of grilled chicken with a side salad (lettuce, cucumber, tomato)
- A large steak and fried eggs
- Not sure

**Q1.6 Which is a better recovery meal option for an athlete who wants to lose weight?**

- A side salad with no dressing (lettuce, cucumber, tomato)
- A pure whey protein isolate (WPI) shake made on water
- A mixed meal that includes a small-moderate serving of meat and carbohydrate (e.g., small bowl pasta with lean mincemeat and vegetable sauce) plus a large side salad
- Not sure

# Macronutrients

**Q2.1 In the early recovery phase after a period of intense training or competition, athletes should aim to consume….**

- 1 – 1.2 g carbohydrate per kg body weight per hour in the first 4-6 hours
- 5-7 g, per kg body weight per hour within 24 hours
- 25% of total daily carbohydrate intake within 2 hours
- Not sure

**Q2.****2 Which of the following options would provide enough carbohydrate to an athlete recovering from about 1 hour of high intensity aerobic exercise? Assume the athlete weighs about 70kg and has an important training session again tomorrow.**

|  | Enough | Not enough | Not Sure |
| --- | --- | --- | --- |
| 1. 1 medium banana (NE) |  |  |  |
| 2. 1 cup cooked quinoa and 1 tin tuna (NE) |  |  |  |
| 3. 1 cup plain yoghurt (NE) |  |  |  |
| 4. 1 cup baked beans on two slices of bread (E) |  |  |  |

**Q2.3 Which food has the most carbohydrate?**

- 1 cup (168 g) boiled rice
- 2 slices of white sandwich loaf bread
- 1 medium (150 g) boiled potato
- 1 medium (150 g) ripe banana
- Not sure

**Q2.4 Do you agree or disagree with these statements about fat?**

|  | Agree | Disagree | Not Sure |
| --- | --- | --- | --- |
| 1. The body needs fat to fight off sickness (A) |  |  |  |
| 2. Athletes should not eat more than 20g of fat per day (D) |  |  |  |
| 3. When we increase the intensity of exercise, the % of fat we use as a fuel also increases (D) |  |  |  |
| 4. When we exercise at a low intensity, our body mostly uses fat as a fuel (A) |  |  |  |

**Q2.5 Do you think these foods are high in fat?**

|  | Yes | No | Not Sure |
| --- | --- | --- | --- |
| Cheddar cheese (Y) |  |  |  |
| Margarine (Y) |  |  |  |
| Mixed nuts (Y) |  |  |  |
| Honey (N) |  |  |  |

**Q2.6 Do you agree or disagree with the statements about protein?**

|  | Agree | Disagree | Not Sure |
| --- | --- | --- | --- |
| 1. Protein is the main fuel that muscles use during exercise (D) |  |  |  |
| 2. Vegetarian athletes can meet their protein requirements without the use of protein supplements (A) |  |  |  |
| 3. The body has a limited ability to use protein for muscle protein synthesis (A) |  |  |  |
| 4. A balanced diet with enough kilojoules/calories (energy) has enough protein for most athletes (A) |  |  |  |

**Q2.7 Which food has the most protein?**

- 2 eggs
- 100g raw skinless chicken breast
- 30g almonds
- Not sure

**Q2.8 The protein needs of a 100 kg well trained resistance athlete are closest to:**

- 100g (1g/kg)
- 150g (1.5g/kg)
- 500g (5g/kg)
- They should eat as much protein as possible
- Not sure

**Q2.9 Which of these foods do you think have enough protein to promote muscle growth after a bout of resistance exercise?**

|  | Enough | Not enough | Not Sure |
| --- | --- | --- | --- |
| 1.100g (3 ounces) chicken breast (E) |  |  |  |
| 2. 30g (1 ounce) Yellow cheese (NE) |  |  |  |
| 3. 1 cup baked beans (NE) |  |  |  |
| 4. 1/2 cup cooked quinoa (NE) |  |  |  |

**Q2.10 Do you think these foods have all the essential amino acids needed by the body?**

|  | Yes | No | Not Sure |
| --- | --- | --- | --- |
| Beef steak (Y) |  |  |  |
| Eggs (Y) |  |  |  |
| Lentils (N) |  |  |  |
| Cow's Milk (Y) |  |  |  |

**Q2.11 The amount of protein in skim milk compared to full cream milk is:**

- much less
- about the same
- much more
- Not sure

# Micronutrients

**Q3.1 Do you agree or disagree with these statements on vitamins and minerals?**

|  | Agree | Disagree | Not Sure |
| --- | --- | --- | --- |
| 1. Calcium is the main component of bone (A) |  |  |  |
| 2. Vitamin C is an antioxidant (A) |  |  |  |
| 3.Thiamine (Vitamin B1) is needed to take oxygen to muscles (D) |  |  |  |
| 4. Iron is needed to turn food into usable energy (D)  5. Vitamin D enhances calcium absorption (A)  6. Meat, chicken and fish are good sources of zinc (A)  7. Wholegrain foods are good sources of vitamin C (D)  8. Fruit and vegetables are good sources of calcium (D)  9. Fatty fish is a good source of vitamin D (A)  10. Women who have a monthly period need more iron than men (A)  11. Athletes aged 15 to 24 years need 500 mg of calcium each day (D)  12. A fit person eating a balanced diet can improve their athletic performance by eating more vitamins and minerals from food (D)  13. Vitamins contain energy (kilojoules/calories) (D) |  |  |  |

# Sports Nutrition

**Q4.1 Athletes should drink water to:**

- keep plasma (blood) volume stable
- stop dry mouth
- allow proper sweating
- All of the above
- Not sure

**Q4.2 Experts think that athletes should:**

- drink 50 - 100 ml (1.7 - 3.3 fluid ounces) every 15 - 20 minutes
- suck on ice cubes rather than drinking during practice
- drink sports drinks (e.g., Powerade) rather than water when exercising
- drink to a plan, based on body weight changes during training sessions performed in a similar climate
- Not sure

**Q4.4 How much fluid should be consumed for rehydration purposes after exercise**

- At least 1.0 – 1.25 L per kg body weight lost
- At least 3-5 L per kg body weight lost
- None
- Not sure

**Q4.5 Before competition, athletes should eat foods that are high in:**

- fluids, fat and carbohydrate
- fluids, fibre and carbohydrate
- fluids and carbohydrate
- Not sure

**Q4.6 Do you agree or disagree with the statements on carbohydrate?**

|  | Agree | Disagree | Not Sure |
| --- | --- | --- | --- |
| 1. Eating carbohydrates when you exercise makes it harder to build strength and muscles (D) |  |  |  |
| 2. In events lasting 60 - 90 minutes, 30- 60 g (1.0 - 2.0 ounces) of carbohydrates should be eaten per hour (A) |  |  |  |
| 3. Eating carbohydrates when you exercise will help keep blood sugar levels stable (A) |  |  |  |

**Q4.7 Some athletes get a sore stomach if they eat during exercise. What might make stomach pain worse?**

- Having energy gels rather than water or sports drinks
- Having small amounts of water at a time
- Having sports drinks with different types of carbohydrates (e.g., fructose and sucrose)
- Not sure

**Q4.8 During a competition, athletes should eat foods that are high in:**

- Fluids, fibre and fat
- Fluids and protein
- Fluids and carbohydrate
- Not sure

**Q4.9 Which is the best snack to have during an intense 90-minute training session?**

- A protein shake
- A ripe banana
- 2 boiled eggs
- A handful of nuts
- Not sure

**Q4.10 After a competition, athletes should eat foods that are high in?**

- Protein, carbohydrate and fat
- Only protein
- Only carbohydrate
- Carbohydrate and protein
- Not sure

**Q4.11 How much protein do you think experts say athletes should eat after resistance exercise?**

- 0.3g/kg body weight (~ 15 - 25 g for most athletes)
- 1.0 g/kg body weight (~ 50 - 100 g for most athletes)
- 1.5g/kg body weight (~ 150 – 130 g for most athletes)
- Not sure

# Supplementation

**Q5.1 Do you agree or disagree with the statements about vitamin and mineral supplements?**

|  | Agree | Disagree | Not Sure |
| --- | --- | --- | --- |
| 1. Vitamin C should always be taken by athletes (D) |  |  |  |
| 2. B vitamins should be taken if energy levels are low (D) |  |  |  |
| 3. Salt tablets should be taken by athletes that get cramps when they exercise (D) |  |  |  |
| 4. Iron tablets should be taken by all athletes who feel tired and are pale (D) |  |  |  |

**Q5.2 All supplements are tested to make sure they are safe, don’t have any contamination.**

- Agree
- Disagree
- Not sure

**Q5.3 Supplement labels may sometimes say things that are not true.**

- Agree
- Disagree
- Not sure

**Q5.4 Do you agree or disagree with the statements about supplements?**

|  | Agree | Disagree | Not Sure |
| --- | --- | --- | --- |
| 1. Creatine makes the brain think that exercise feels easier (D) |  |  |  |
| 2. Caffeine makes muscles able to work harder even without more oxygen (D) |  |  |  |
| 3. Beetroot juice (nitrates) makes muscles feel less sore after exercise (D) |  |  |  |
| 4. Beta-Alanine can decrease how much acid muscles make during intense exercise (A)  5. Supplements containing ferulic acid can increase lean body mass (D) |  |  |  |

**Q5.6 WORLD ANTI-DOPING AGENCY (WADA) bans the use of….**

- caffeine
- bicarbonate
- carnitine
- testosterone
- Not sure

# Alcohol

**Q6.1 How much ethanol (pure alcohol) is there in a standard drink?**

- 1 – 2g
- 8 - 14 g
- 30 - 50 g
- Not sure

**Q6.2 Which is an example of a "Standard Drink"**?

- 25 ml of pure spirits
- One quarter of a bottle (175 ml) of red wine
- A pint (425 ml) of full-strength beer
- Not sure

**Q6.3 Do you think alcohol can make you put on weight?**

- Yes
- No
- Not sure

**Q6.5 Do you agree or disagree with the statements on alcohol?**

|  | Agree | Disagree | Not Sure |
| --- | --- | --- | --- |
| 1. If someone does not drink at all during the week, it is okay for them to have five or more drinks on a Friday or Saturday night |  |  |  |
| 2. Drinking lots of alcohol can make it harder to recover from injury |  |  |  |
| 3. Alcohol makes you urinate more |  |  |  |

**Q6.6 "Binge drinking" (also referred to as heavy episodic drinking) is defined as:**

- having two or more standard alcoholic drinks on the same occasion
- having four to five or more standard alcoholic drinks on the same occasion
- having seven to eight or more standard alcoholic drinks on the same occasion
- Not sure

***** Questions highlighted were adapted to make more relevant for our population.

**Appendix 3. Interview discussion guide**

| **Q1:** Describe in your own words what you think a healthy diet is – why? Can you give some examples of healthy foods? |
| --- |
| **Q2:** Describe what you think an unhealthy diet is – why? Can you give some examples of unhealthy foods? |
| **Q3**: Do you think your diet influences your performance in any way? Describe nutritional strategies you apply |
| **Q4**: What aspects of nutrition in particular, if any do you think, influence how you perform? Why? |
| **Q5**: Are there any specific foods, drinks, or products that you take to help enhance your performance? How do you feel these benefit you? |
| **Q6**: How do you think the timing of meals is relevant to you as an athlete? |
| **Q7**: What are your main dietary goals as an athlete? |
| **Q8**: At what times of year do you ensure nutrition is a priority? |
| **Q9**: Are there any other factors that we have not discussed that influence what or how you eat? (e.g., Time, money, skills, media, taste, convenience, ergogenic aids, education, availability) |
| **Q10:** What is the biggest challenge in terms of meeting your dietary goals these days? (Hint for researchers: current climate impact on players) |
| **Q11:** Do you feel that you are struggling to meet specific goals? (Hint for researchers: current climate impact on players) |
| **Q12:** What impact do you feel this is going to have on your body and/or performance? |
